# Using the Maximal Entropy Modeling Approach to Analyze the Evolution of Sedentary Agricultural Societies in Northeast China

**DOI:** 10.3390/e22030307

**Published:** 2020-03-09

**Authors:** Ido Wachtel, Royi Zidon, Gideon Shelach-Lavi

**Affiliations:** 1 The Institute of Archaeology, the Hebrew University of Jerusalem, Jerusalem 9190501, Israel; 2 Department of Evolution, Systematics, and Ecology, the Hebrew University of Jerusalem, Jerusalem 9190501, Israel; zidonr@gmail.com; 3 Department of Asian Studies, the Hebrew University of Jerusalem, Jerusalem 9190501, Israel

**Keywords:** maximal entropy, MaxEnt, northeast China, locational modelling, Neolithic, transition to agriculture, settlement patterns

## Abstract

The emergence of agriculture and the evolution of sedentary societies are among the most important processes in human history. However, although archeologists and social scientists have long been studying these processes, our understanding of them is still limited. This article focuses on the Fuxin area in present-day Liaoning province in Northeast China. A systematic archeological survey we conducted in Fuxin in recent years located sites from five successive stages of the evolution of agricultural sedentary society. We used the principles of Maximal Entropy to study changes in settlement patterns during a long-term local trajectory, from the incipient steps toward a sedentary agricultural way of life to the emergence of complex societies. Based on the detailed data collected in the field, we developed a geo-statistical model based on Maximal Entropy (MaxEnt) that characterizes the locational choices of societies during different periods. This combination of high-resolution information on the location and density of archeological remains, along with a maximal entropy-based statistical model, enabled us to chart the long-term trajectory of the interactions between human societies and their natural environment and to better understand the different stages of the transition to developed sedentary agricultural society.

## 1. Introduction

The transition to agriculture (including the domestication of plants and animals and the development of a sedentary way of life) was arguably the most significant ‘revolution’ in human history [1,2]. This transition gradually led to a dramatic increase in population size and density, in craft specialization and the division of labor, to the initiation of social dynamics and the accumulation of resources that are linked to the development of socio-political stratification, and finally, to the appearance of large regional states. Research on the transition to agriculture and its short and long term effects has been high on the agenda of archeologists since the early days of our discipline [1,3]. However, our understanding of the dynamics of these processes is still incomplete. For example, contrary to earlier models, which described the beginning of agriculture as an abrupt event [4], current archeological and ethnographic research contests the clear-cut dichotomy between hunter-gatherer and agriculturalist societies and describes the transition process as long and variable [5,6,7]. The development of social complexity is, furthermore, viewed not as a unilineal process which evolved along a similar trajectory around the world, but rather as a much more complex multi-variable process that can evolve along many different pathways [8,9]. Therefore, in order to better understand those fundamental process in human history, it is crucial to locate them in the specific ecological and socio-cultural contexts of their occurrence in different parts of the world. 

In this paper, we are focusing on the analysis of data from northern China, the second oldest center of plants and animal domestication in the Old World and a region where the transition to agriculture led to the emergence of unique civilizations and the development of complex societies and states [10]. We focus on a specific sub-region within this large region, that of Northeast China, in an attempt to flesh-out the ecological basis for the socio-economic transformation that occurred there from the incipient transition to agriculture and sedentary life-ways to the formation of complex societies. Our focus is on environmental characteristics that govern the selection of site location during different phases of the long-term trajectory in this region. Focusing on site location as an indication of the decision making of prehistoric societies is not a new idea. However, using high-resolution data and analyzing it within the Maximal Entropy framework allows us to tackle this issue much more rigorously and to generate insights that are not obvious from looking at site distribution maps. Those insights are relevant to our understanding of the local trajectory and can be used in comparisons with other regions in China and world-wide. 

The Maximal Entropy modeling approach was developed and applied to the social sciences beginning in the 1970s, if not earlier. It has been widely used in human geography and urban planning to model spatial interactions [11]. Similar techniques were also used by archeologists, mostly during the last decade, to predict the location of archeological sites and describe the expansion or contraction of settlements within a region [12,13,14]. The Maximal Entropy model (MaxEnt) which is the focus of this paper, is a machine learning algorithm developed for ecologists wishing to model species using presence-only species records [15,16], a situation which is also very common in archeological research. Since its publication in 2004, MaxEnt has rapidly become the dominant modeling approach for species distribution thanks to its high performance [17]. In recent years, it was also adapted for archeological locational modeling [18,19,20,21,22]. However, MaxEnt is still not widely used in archaeology, where it has great potential to advance our knowledge of early societies. 

The archeological locational (predictive) model is a tool that helps to assess the probability of sites being present in different locations in the landscape [23,24,25]. At the same time, it helps to analyze and understand the ways in which people used their environment through time. Thus, these models are essential for answering key anthropological questions [26,27,28,29]. Although locational modeling tools have been used in archeology for decades, they still suffer from several basic problems (both methodological and technical) as a cultural interpretive tool [25,30]. One main argument is that locational models are not accurate enough (in both their data sets and statistical procedures), while another is that cultural phenomena should not be explained based on deterministic models founded upon partial, and sometimes biased, data sets [31]. It is also argued that archeological locational models often rely on unsystematic surveys and that their sample is biased. Another issue is related to the need of such models for site-presence and site-absence data-sets, while in archeology the site-absence is usually unknown. The great advantage of the data presented in this paper is that it is based on a systematic full-coverage survey. We are therefore certain that all (or at least the vast majority) of sites that have visible remains on the surface were identified and documented by us. Use of the MaxEnt model, which is based on presence only data (site-presence), together with its ability to overcome autocorrelation among predictor variables [15,16,17] is expected to overcome this type of problems.

In a previous paper [22], we compared the conventional statistical approach to locational/predictive modeling with MaxEnt. Our main conclusion was that MaxEnt has the ability to provide stable and effective results based on small and partial archeological data set. This type of analysis and accuracy was not previously possible in archeological locational modeling, which is often based on a limited number of observations. In the current paper we take this approach one step forward and use the MaxEnt algorithm to analyzed location preferences (habitats or ecological niches). Rather than asking where new sites should be found based on the location of known sites, we ask what are the ecological and geographical parameters that define the location of the sites we know. We then compare the results of this analysis of sites from different subsequent periods, which were accurately recorded and chronologically defined by the Fuxin systematic archeological survey [32,33,34]. We are using the same research area and dataset from our previous paper, but in this study we apply the model to each archeological period separately, in order to distinguish between different locational strategies throughout the different stages in the evolution of sedentary society throughout the Neolithic and early Bronze Age periods. Two basic research questions are derived from this framework, which are methodological and anthropological: Methodologically, is the MaxEnt model effective in identifying different location patterns and distinguishing between different periods, even when using a small database of 15–20 observations per period? Anthropologically, is it possible to identify a process of changes in the locational preferences of ancient societies throughout the different phases of the prehistoric trajectory of the Fuxin area? Or, in other words, what are the spatial manifestations of economic and social changes throughout this trajectory?

### 1.1. The Fuxin Regional Survey: Methods and Results

**Figure 1:**
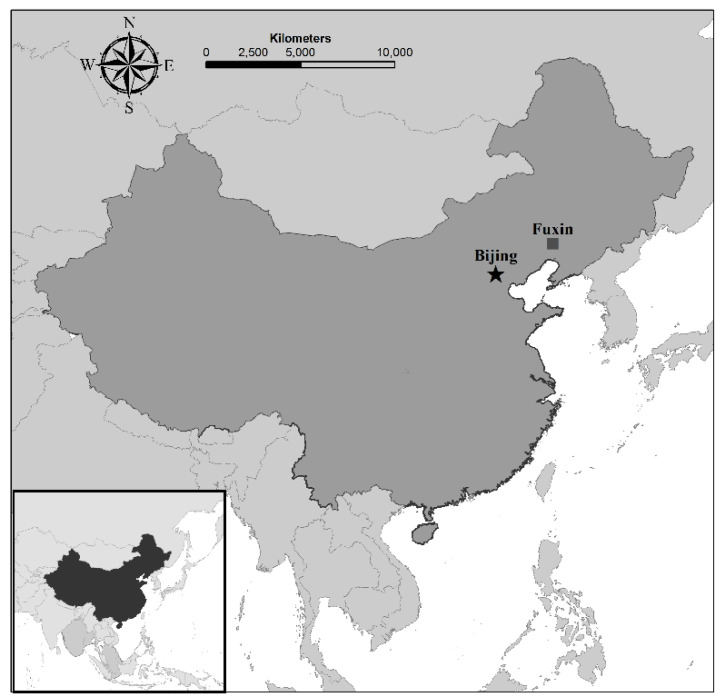
Location of the Fuxin area in China.

Our archeological research in the Fuxin area of western Liaoning Province (Figure 1), was a collaborative project that brought together archeologists and students from the Research Center for Chinese Frontier Archaeology at Jilin University, the Hebrew University of Jerusalem, Ben-Gurion University of the Negev, and the Liaoning Provincial Institute of Archaeology and Cultural Relics. The project examined the long-term trajectory of socio-economic changes in this region, starting from the incipient development of agriculture and sedentary ways of life and continuing to the developments of more densely populated complex societies. This trajectory spans a period of roughly five thousand years, from c. 6000 BCE to c. 1200 BCE. Our project included systematic regional surveys [32,35] as well as test excavations at sites located during our survey [33,34,36]. All the data from the Fuxin regional project is openly available [37]. 

In this paper, we use the data collected during two seasons of systematic regional survey (2012 and 2013), where we were able to cover some 104 sq·km. During the survey, team members walked close to one another (20 m·spaces between one person and the next) and were instructed to walk slowly and pay careful attention even to small and very sparse concentrations of artifacts. We paid special attention to stone artifacts, which have not been identified and systematically collected in other surveys in China. Survey conditions in the Fuxin area are close to ideal, as the area occupied by modern villages and other man-made features comprises less than 10% of the entire region. Ground visibility during the time of year that the Fuxin survey was conducted (March and April) were excellent: the ground in the fields and orchards was completely exposed, allowing for extraordinary visibility of archeological artifacts. Consequently, we were able to identify and document many sites that were previously unknown. Locating sites inside the forested areas was more difficult, but using intensive sampling methods we were able to identify and rigorously assess the density of prehistoric human occupation in this ecologic niche as well [32]. 

When the survey group encountered ancient artifacts or features, their location was recorded using GPS technology, and a sample of the artifacts was collected to identify their date and density on the ground. Rather than “sites” which may be more vaguely defined or include several collections from different periods, we used those “collections” as the primary analytical unit. The GIS analysis discussed below is based on the location, dimension, artifact dates, and the density of artifacts recorded for each “collection”. All together, we recorded 1152 collections, some containing evidence of occupation during more than one period. The basic information related to the five periods addressed by this paper are presented in Table 1 and the detailed data on which it is based is presented in the Supplementary data (S2). While the variability among collection units is usually not that large in some periods, most notably during the Bronze Age, collection units tend to cluster together, forming what more traditional accounts call a “site”. Because our analysis considers each collection unit as an independent observation, such clustering affects the analysis and reflects the variability of our survey results. 

### 1.2. Research Hypothesis

The periods of occupation included in this study represent the trajectory from the beginning of sedentary lifeway in the Fuxin region, through the advancement of complex agricultural societies, to the development of more densely populated polities (chiefdoms?) and larger central sites (cities?). It started in the Xiaohexi period (c. 6000–5700 BCE), during which small scale sites with minimal investment in permanent structure may represent the incipient settling down of groups of complex hunter-gatherer societies [36]. During the ensuing Xinglongwa period (Xinglongwa c. 5700–5200 BCE) we see a rapid transition to relatively large-scale villages, which accompanies the development of agriculture. However, our recent research suggests that even during this time, wild plants and animals were the dominant resources utilized by the Xinglongwa population, with domesticated foods only accounting for a small portion of the economy. The evolution of village life continued through the Zhaobaogou period (Zhaobaogou c. 5200–4500 BCE) [38] and peaked during the Late Neolithic or Hongshan (Hongshan c. 4500–3000 BCE) period. The Hongshan period is well known for its evolved Jade industry and unique ritual centers, but such centers have so far not been found in the Fuxin region [39]. Following a period of about a thousand years of depopulation (or even settlement hiatus), the early Bronze Age societies of this region, known as the Lower Xiajiadian culture (Lower Xiajiadian c. 2000–1200 BCE), emerged to present one of the peaks of regional population density with the construction of labor-intensive fortifications, a high quality ceramic industry, and the incipient production of small bronze artifacts [40,41].

The Fuxin regional archeological study focused on processes of socio-political and economic change. An important issue we address in the present article is how those processes are reflected in the interaction between human communities and their environment. More specifically, we want to know how people choose where to locate their community (village) and how this choice changed in accordance with changes in the social and economic organization. Among the environmental factors that are relevant to such an analysis are limiting conditions, such as steep slopes and exposed terrains, as well as attractive resources, such as water, arable land, and natural sources of food and raw materials. 

We know that significant climatic changes occurred during the 5000 years period addressed by this paper: the climate improved dramatically (more rain and warmer temperatures) around 5800 BCE, more or less coinciding with the beginning of the Xinglongwa period, and it worsened during the late 4th millennium BCE, more or less coinciding with the decline of the Hongshan society [36]. While these changes may have affected the development of human society, we argue that they are not very relevant to our understanding of the environment and of human interaction with it. While some parameters, which we cannot at this point evaluate, may have changed, the parameters used here, such as the location of main rivers, slopes, arable land, and the like, remain the same regardless of decreases or increases in precipitation or temperatures, and their attractiveness or lack of attractiveness is related to human behavior, technology, and social organization more than to climatic conditions. 

Our initial working hypothesis was that, with the progression of time, collection units (and their clusters or ‘sites’) will become more closely associated with environmental elements that are crucial for agriculture and its intensification—elements such as arable and fertile land, stable water resources and good exposure to sunlight. Thus, we expected that Xiaohexi collections would be the least associated with such parameters and more closely associated with conditions that are good for hunting and gathering, and possibly also with locations with an abundance of stones suited to the production of stone tools. We expected the subsequent period of Xinglongwa, and even more so, the Zhaobaogou and Hongshan collections, to be closely associated with environmental niches best suited for agriculture. The Lower Xiajiadian collections of the Bronze Age are also expected to be associated with such environments but, because some of the sites are very large (an aggregate of many collectionsunits) and are fortified, other considerations such as the selection of large areas that are easily defendable, might affect the overall picture. Analyzing the location of collection units found by our survey using geo-statistical models that are based on Maximal Entropy enabled us not only to test those simple hypotheses, but to flesh out more fully the complex decision making processes and the factors involved in such decisions made by the prehistoric populations.

## 2. Materials and Methods 

In previous work, we used the results of the Fuxin Survey to predict the potential location of sites in a wider neighboring area. The geographical framework of the current model overlaps with the Fuxin survey area, aiming to use the model as an interpretative tool for the characterization of the ecological niches typical for each period and an examination of changes over time. The model area is 104 sq·km. It was divided into 10,400 cells, each measuring 100 × 100 m. The model’s purpose is to predict, for each such cell, the relative probability of collection unite presence, based on the different environmental parameters of each cell. Because the location of the sites (represented here by the collection units) is known definitively from the results of the systematic survey, the model results are used to explain the preferred ecological niches of each period. The dependent variable in the model is an archeological collection unit of a specific period, while the independent variables are various environmental datasets that were determined based on the geographic information available for the survey region and on our observations during the survey itself (see S1 for data sets and code scripts).

### 2.1. Datasets

The archeological dataset: In the Fuxin survey, 1152 collection units were recorded within a total area of 746.3 hectares. Of these, 126 collection units (56.5 hectares) included pottery shards dating to different stages of the Neolithic period and an additional 105 collection units (60.8 hectare.) were dated to the first phase of the Bronze Age (the LXJD period), (Table 1). For each survey collection unit (polygon) we created a centroid which includes the coordinates and the archeological data. The Fuxin Survey datasets are available online, in the Comparative Archaeology Database on the University of Pittsburgh website [37].

The environmental dataset: The relevant environmental variables included in the models were selected based on well-established hypotheses regarding the type of factors which affected human decision making in northeast China [32,35,42]. Those variables include:

Topographic variables: slope, aspect and land curvature: We used 12.5 m satellite imagery from the Japanese Aerospace Exploration Agency (JAXA) available from the ASF DAAC datasets (https://search.asf.alaska.edu/#/). This imaging contains elevation data, which enabled us to create raster layers of slopes, aspect, and local terrain (land curvature). These variables are fairly common in archeological predictive models, based on the logical assumption that people tended to live in moderate areas [43]. The land curvature parameter was produced using the GIS’s curvature function (ArcGIS 10.4 by ESRI). This function calculates and highlights different aspects of the shape of the slope, and presents the differences between each cell and its neighbors (sometimes defined as “the slope of the slope”). Aspect is another common variable in location models in different parts of the world [43,44]. In cold climates (such as Northeast China), for example, there is a preference for locating habitation sites on slopes with more sun exposure (south-facing slopes in the northern hemisphere), which receive more sunlight and heat during the day. 

Proximity to water sources: Proximity to water sources is a significant factor in the existence of a settlement, especially in arid and semi-arid regions. In the Fuxin area, the three main rivers which cross the study area appear to have been the primary source of water for human habitation throughout history. While they may freeze over in the winter, these rivers nonetheless supply fresh water throughout most of the year. In both study areas we constructed a raster layer of 100 × 100 m using the Euclidean distance method, which calculates the distance between each pixel and the closest water source (a spring in north Israel or a river in Northeast China). 

Modern land uses: Because of the advent of agriculture and cultivation techniques, the current use of the land does not precisely reflect the way it was used in the past. Nonetheless, current land use can be employed as a heuristic device that suggests a proxy, even if its nature is not precisely known, to the productivity of the land and thus, to its attractiveness for humans. Here we used a Euclidean distance function of the present-day land uses: agriculture fields, forests, and modern settlements. We produced vector layers based on QuickBird orthophoto (1:50,000 scale) for modern built areas, crops areas, and forests. Then we calculated the Euclidean distance of each survey collection unit in each layer, using the GIS “near” function. 

### 2.2. Methods

The study area was divided to 10,400 squares of one hectare (map pixels of 100 × 100 m) each. The MaxEnt algorithm calculates relative probability for each 100^2^ cell/pixel, which can basically be presented as a gradual color map of continuous values. The probability values were then projected as a binary map (high probability / low probability) using a threshold value determined according to model performance. For the threshold of high/low probability, we chose the actual probability value for each period model which is able to “predict” 85% of the collection units in the dataset. This threshold and the use of a binary map rather the actual model’s values, is widely accepted in archeological locational modeling [25,43]. However, in order to avoid the arbitrariness of this threshold, we used additional thresholds of 80%, 85%, and 90% for each period model. Our discussion is based on the 85% threshold but, for comparative purposes, the model results of the thresholds that predict 80% and 90% of each period’s collection units are also presented. Since MaxEnt returns the probability of a cell containing a settlement, a threshold must be set so it can be converted into a binary map. The choice of a threshold depends on whether one wishes to minimize false negative or false positive errors. A threshold that is too high will result in a high number of false negative errors (settlements outside the positive area), leading to a small cover area. Conversely, a threshold that is too low will result in fewer false negatives and a higher cover area. We selected a threshold by setting the false negative rate at 0.15, which means 15% of the known settlements will be outside the positive area 

In our case, we already know where sites exist from the systematic survey. In this sense, we are using the model as explanatory tool, which helps us to understand and interpret the locational preferences of different periods, as well as identify a general pattern of such preferences. The model results (areas with high probability) can be interpreted as habitats, or the ecological niches that are most suitable for each of the cultural phases under investigation. The areas returning a high probability are those that are most suitable for settlement sites, based on the analysis of our observations (collection units) and their environmental properties (background variables).

### 2.3. Model Fit and Effectiveness 

There are a number of widely accepted approaches to examining the effectiveness of a given model and its performance. The most common approach requires that the model should predict at least 80–85% of the collection units located in about 33% of the land defined as “high probability” areas. The smaller the predicted area is (33% or less), and the higher the percentage of collection units located in it (80% percent or more), the more effective the model is considered to be [25,45]. We used this approach as a starting point for each of the models we built. The threshold value for the binary maps (high / low probability per collection unite presence) would thus be a value that efficiently and accurately predicts 85% of our observations (collections). In this way, the high probability model area (henceforth: land cover) expresses the model’s relative success. We added two common statistic indicators to these test: area under the curve (AUC) [46] and Test gain [47], both of which are common measures of model effectiveness. 

We used three indicators to assess how well the model discriminates between unsuitable and suitable areas. These indicators are the AUC, Test Gain, and the ‘cover area’. The AUC score is a common statistic tool to assess and compare models [48]. It represents the probability that a randomly chosen presence collection unit will be ranked as more suitable than a randomly chosen pseudo-absence collection unit. At random, the AUC value would be ± 0.5, whereas a model with good discrimination would have a higher AUC. The test gain describes how much better the MaxEnt model distribution fits the presence data as compared to a uniform distribution [49]. The higher the value, the better the model discriminates between unsuitable and suitable areas. The ‘cover area’ is the size of the area that is classified as the area suitable for finding settlements, divided by the total research area size. The smaller the model area is, while still predicting 85% of the collection units, the more effective the model is considered. This is true here for each model period, and for the understanding of each variable’s importance to the model (see below). 

We are fully aware of the procedure of cross-validation based on independent samples of both training and testing datasets [21,23,43]. However, in the current study, the sample group of collection units is a priori very small, and this sample size does not allow for an effective control group. Alternatively, we used 5-fold cross-validation for each set of data (different periods). In each fold we used 85% of the collection units. The XLW and LXJD periods have many more collection units (79 and 104, as compared to 20 on average for the other periods). In order to verify that the difference in size did not affect the model’s performance, we ran the model with 85% of the collection units and 25% of the collection units. 

### 2.4. Understanding Variable Importance

Because the purpose of this study is to explain the environmental considerations of groups at different stages of permanent settlement and agriculture, we undertook further analysis to show which environmental variables are most relevant to the model during each period. In order to understand the importance of each variable, we used the jackknife method [47]. After running the model with all the variables, we ran the model again, with all the variables except for one, and then we ran the model with just that one variable. Using model performance indicators, we analyzed, for each run, the land cover, test gain, and AUC [21,47]. This comparative approach between parameters allowed a statistical distinction between the contribution of each parameter alone to the general model: the smaller the model area, and the higher the test gain that the parameter contributed to the general model (by running a discrete variable). Autocorrelation among predictor variables may be a problem in this type of analysis. To avoid this, we tested autocorrelation between variables. Our results confirm that such correlations are not strong and that do not affect the results of our analysis. See the Supplementary data (S3) for more information.

## 3. Results

Using the MaxEnt model, five successive periods were examined. The location of collection units was analyzed in relation to eight environmental categories. The categories include: Distance from the main rivers (River Dist.); the degree of slope (Slope); the aspect of the slope (Aspect); elevation above sea level (Elevation); Land Curvature, a GIS function which calculates the shape of the slope (this is most useful for identifying areas with rapid change in a slope or aspect) [50]; distance from land that is currently used for agriculture (Crop Dist); distance from land that is currently forested (Forest Dist.); and distance from modern villages (Modern Built Distance). Because it is impossible to obtain high resolution geologic maps or maps of soils and land uses for China, we had to use parameters that we could extract from satellite images and verify them in the field. While some of these parameters are based on modern conditions that did not exist during the prehistoric period, we use them as heuristic devices that mark certain types of environments. For example, although the forests were all planted during the past 30 years, they are located on high rocky and steep ground that was not suitable to any other purposes. Thus, their location marks a specific zone and the distance from it may have economic significance.

The results of each period model are summarized in Table 2, and for each model, a binary probability map was prepared. This map expresses high and low probability areas. For each period model, the percentage of “high probability” in the model (henceforth: land cover) expresses the model’s performance while predicting 85% of the period’s collection units. The AUC value and test gain were calculated for each period model, expressing model fit (Table 2, and see Material and Methods below). In other words, the test gain provides an indication of the model’s significance in relation to a random model.

In addition, the most influential environmental parameters for each period were analyzed in order to test our hypothesis regarding the evolution of sedentism and cultivation through the Neolithic and how such socio-economic changes are reflected by both ecological niche / habitat and the different environmental variables. For the XLW and LXJD periods, we ran the model with only 0.25 observations, in order to study the effect of the sample size. No significant effect was found. 

### 3.1. Xiaohexi (XHX)

**Figure 2:**
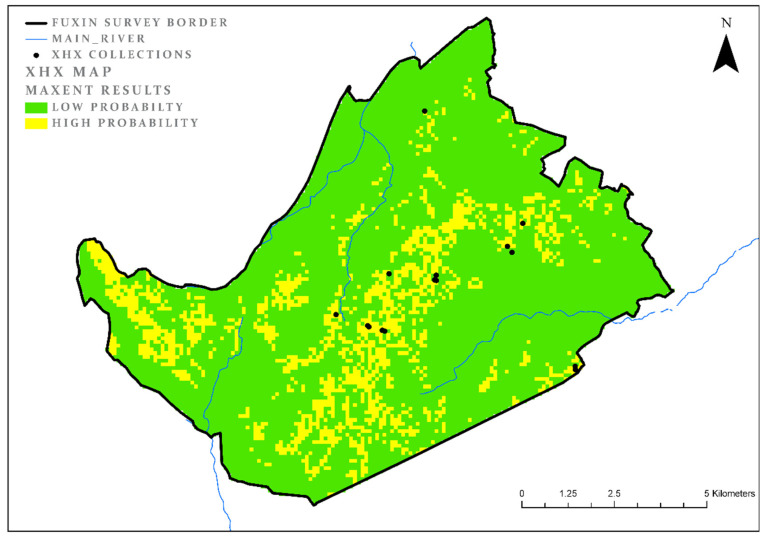
XHX model results.

The first period in our analysis, the *XHX*, is the earliest settlement period discovered in the study area, and the first phase in the transformation to a sedentary society. The model was built on the basis of 15 collections units dated to this phase. The model results for this period comprise 19% of the study area, and reflect the typical preferred ecological niches (or habitats) of this culture (Figure 2). The model’s AUC value is high (0.88) and so is the test gain (1). The most significant environmental parameter for this period is the aspect. Habitation sites from this period in the research area (and probably beyond it as well) tended to prefer a south aspect, for the basic reason that it is more exposed to sunlight and heat than other aspects. The results of the MaxEnt model which was run on this parameter individually include 36.9% of the study area with test gain of 0.36. These by themselves are relatively significant results. Another significant variable is location close to forested areas. As noted above, such locations are not suitable for agriculture, but they may present good areas for hunting and perhaps for obtaining other resources, such as exposed stones suitable for tool production. Using this variable alone, a model can be constructed that will comprise 45.2% of the area, with a high test gain of 0.31 (Table 3). The map (Figure 2) shows that the main areas covered by this model are the mountainous areas, far from the main river basins and the nearby alluvial valleys. These are areas that today are less suitable for cultivation, and probably were in the past as well.

### 3.2. Xinglongwa (XLW)

**Figure 3:**
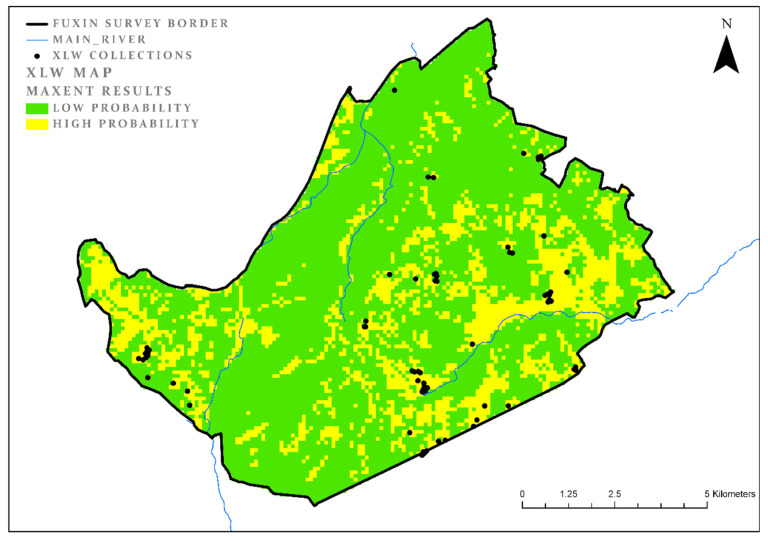
XLW model results.

The subsequent period, XLW, has a larger number of collection units (72). In order to create samples of similar size, 25% of these (a subset of 18 collection units) were randomly sampled, where the results are an average of four model runs. The model results for this period are slightly less clear cut, which probably reflects the greater environmental variation of site locations. The percentage of land cover for the XLW model is 26%, the AUC is 0.82 and test gain is 0.85 (Figure 3). The most significant variable for the XLW period, similarly to the XHX period, is the aspect (land cover = 38%, test gain = 0.39). The second influential parameter is proximity to agricultural lands (land cover = 65%, test gain = 0.25). A look at the map shows that the preferred ecological niches / habitats are split between the central mountain range (similar in a way to the previous period), and areas close to the main rivers and alluvial valleys adjacent to them (Table 4, Figure 3). 

### 3.3. Zhaobaogou (ZBG)

**Figure 4:**
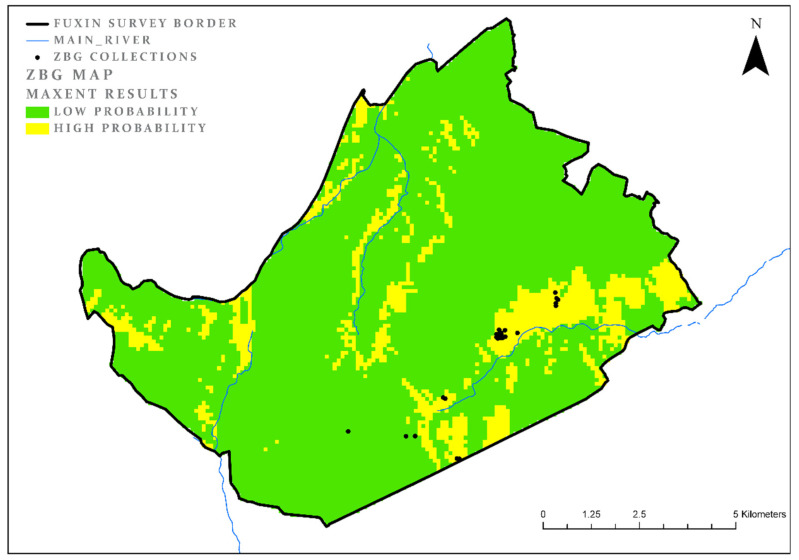
ZBG model results.

For the ZBG period, the survey identified remains in only 24 collection units. The model results during this period cover 14%, of the research area (AUC = 0.93; and the test gain = 2.2). For all the periods we tested, this is the most effective model. It probably indicates a small variation among the location preferences of habitation sites. A look at the map (Figure 4) shows a significant difference from the previous period. While the XLW period presents a preference for both near-river niches and mountainous areas, the ZBG period niches are almost exclusively in the near-rivers areas and lowlands. An examination of the significant parameters (Table 5) shows clear continuity in the importance of the aspect (land cover = 41%; AUC = 0.6) along with proximity to the river (land cover = 45%, AUC = 0.32).

### 3.4. Hongshan (HS)

**Figure 5:**
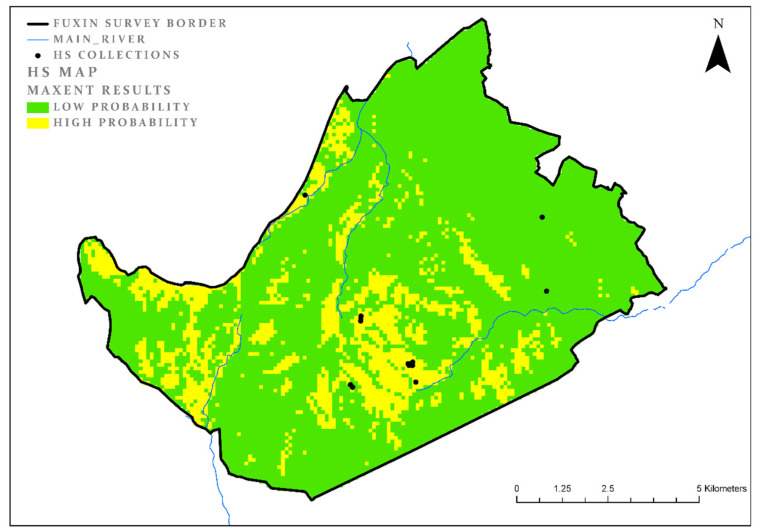
HS model results.

The survey located 15 collection units from the HS period. The model results cover 15% of the study area (AUC = 0.91; test gain = 1.61; Figure 5). The most important environmental parameter remains the aspect (land cover = 49%; test gain = 0.41). However, unlike the previous periods, there is no other parameter for the HS period that is clearly the second most important. Several parameters, including distance from the main rivers, distance from cultivated land, and elevation (sites of this period tend to cluster in slightly higher areas than previous periods) all contribute to the model (Table 6). According to the test gain analysis, each of these parameters contributes to the model more or less equally.

### 3.5. Lower Xiajiadian (LXJD)

**Figure 6:**
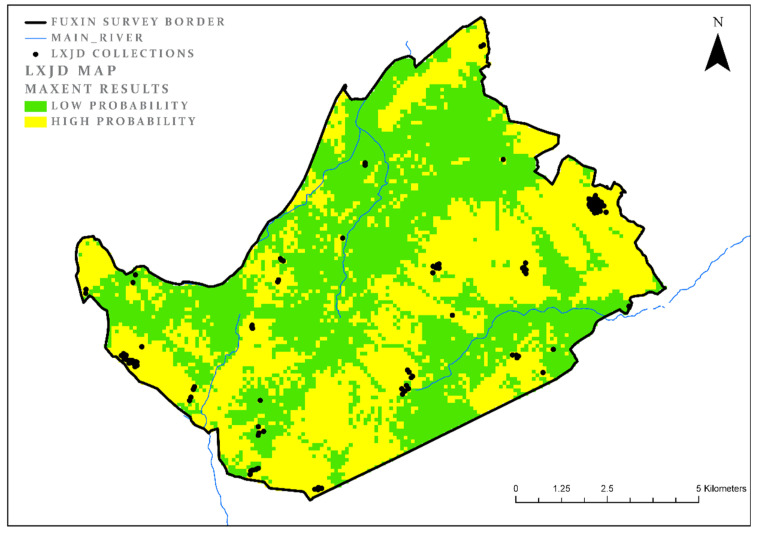
LXJD model results.

As discussed above, after a period of dramatic decrease in settlement density, the LXJD represents the beginning of the Bronze Age in this region. Although bronze production was carried out on a relatively small scale, other aspects of the economy and social composition seem to have changed in comparison to the Neolithic periods. No fewer than 105 collection units were identified for the LXJD, many more than for all the preceding periods, suggesting an increase in the regional population. Here too (like for the XLW period), we used a four-subsets sampling approach with a random selection of 25% of the collections. The model results here are less clear than for the previous periods: The model land cover comprises 37% of the study area, while the AUC measure is only 0.8 (Figure 6). These results suggest a shift in the environmental attributes of the settlement system. Clearly, aspect is still an important factor in the selection of a location in which to settle, but it is no longer the most important one, as it was for all the Neolithic phases. In fact, all the parameters appear to be significant to the model, with each making a similar contribution to the model’s success (Table 7). However, the individual contribution of each parameter is not great, and the model itself is much less effective than the models for the Neolithic periods. One explanation might be that, with the increase in population density, the societies of this period had to choose locations with less than optimal conditions. Yet, even during this period, the area was apparently not very densely populated and many locations were still available, thus it seems that the model we identified reflects an actual selection process in which many parameters were equally important. These include, as indicated in the introduction, not only parameters associated directly with the economic mode of subsistence (agriculture), but also perhaps with political considerations such as the need to select an area which can be easily defended against hostile polities. It is also possible that in order to gain a better understanding of this period we need to include additional parameters relevant to more complex systems, such as, for example, viewshed, visibility, and connectivity. Such work is, however, beyond the scope of this paper.

### 3.6. Different Threshold Values

In Table 8 we present again an analysis of the variables’ importance for each model period, comparing different MaxEnt thresholds, which predicts 80%, 85%, and 90% of the collection units, respectively. The results of these analyses clearly show that there is no significant change in the important variables or in their test gain and land coverage. This result strengthens the model’s robustness. In addition, it should be noted again that the modeling procedure for two of the periods (XLW and LXJD) is based on a sample composed of 25% of observations.

## 4. Discussion and Conclusions

Entropy is a yardstick for disorder and uniformity in a given space. Nature has a tendency to disorder and to random processes, unless some energy is deliberately invested in the opposite direction. This also applies to human location models. We expect uniform dispersal of human settlements in all periods, unless there are forces that distort this random processes. In ancient periods we can assume that there were many limitations, as well as attractions, that altered the random distribution of settlements. As time passes, different constraints and opportunities evolve and change along with the development of human technology and culture. Such changes are reflected in the choice of settlement locations.

Alongside the important insights, it provides regarding the trajectory of socio-economic change in Northeast China, our study also generates important methodological contributions. One key problem in locational modeling in archaeology is the inability to perform a stable model analysis on a small dataset of observations, a situation which is quite common for archeological projects. This is one reason why locational modeling of different sub-periods, or site types is quite rare, as well as why their results are often insufficient. In this study, we demonstrate that a combination of high-resolution accurate survey data and the use of MaxEnt analysis, can provide meaningful results even when working with small datasets. The results of this paper highlight the benefits of an Entropy-based model over previous statistical approaches in archeology (such as logistic regression). As we demonstrated, it is useful not only as a predictive model, for which such techniques are often used (and see discussion in Wachtel et al. 2018) [22], but also as a way to explain the behavior and decision making of prehistoric societies. The ability to create a stable model based on small dataset of observation, and the ability to analyze and rate different parameter relationships for these small datasets is unique to this algorithm. This highlights that MaxEnt is much more suitable for the analysis of archeological data than other predictive modeling approaches, which requires a set of pseudo-absence locations (as non-sites). The entropy-based algorithm forestalls one of the most common problem in archeological locational modeling, because this type of information (non-sites) is usually not readily available to the archaeologist.

Three of the periods we analyzed in this study consist of only 15 to 20 collection units (independent observations), but their analysis resulted in the creation of strong models. The model thus created helped to characterize the way in which the major environmental parameters affected the location of prehistoric communities in each period, as well as the transformation over time in these location strategies. 

From an anthropological perspective, the locational model facilitates a systematic analysis of change in locational preferences over time, and sheds light on human strategy and decision making. Overall, the results support our initial hypotheses about the nature of each phase in the local trajectory, but using the model allowed us to gain a better and more nuanced understanding. At the incipient phase of the trajectory, the Xiaohexi period, the main environmental parameters that affected site locations were the aspect of slopes (preference for southward facing slopes) and proximity to areas which are currently forested. As discussed above, these elevated and rocky areas were not suitable for agriculture, but provided good resources for hunting-gathering, including sources of raw materials for the production of stone tools. Our analysis suggests that the location preferences of these prehistoric societies changed with the transition to the more advance phases of the Neolithic period. Although southward facing slopes (aspect) were favored in all periods, as can be expected from societies in northern latitudes, the later Neolithic societies show a clear tendency to locate their sites near areas that are presently used for agriculture and areas close to the main rivers. In contrast to the Xiaohexi, the location of sites dated to the Xinglongwa, Zhaobaogou, and Hongshan periods is what we would expect from societies whose economic focus is on cultivation. 

As discussed above, our analysis of the location of Bronze Age (LXJD) collections was less successful than that of the earlier periods. The model itself is far less effective (land cover of 37% versus 14–26% in different phases of the Neolithic), and none of the variables contribute significantly to the model (although all the parameters have p.v. < 0.05). Although the model by itself does not sufficiently explain the location strategies of the Bronze Age societies, it does clearly indicate a change from the strategies of the Neolithic societies that previously inhabited the same area. It suggests that Bronze Age societies may have had different considerations, which are not well-reflected in our environmental proxies; nonetheless, a more detailed locational analysis of this period is beyond the scope of the present paper. 

## Figures and Tables

**Table 1:** Summary of the survey results for prehistoric periods (c. 6000–1200 BCE). See additional information on the variability of those results in S2.

Period	Years (BCE)	Number of Collections	Number of Pottery Sherds	Total Area (Hectare)
Xiaohexi (XHX)	6000–5700	15	288	6.724
Xinglongwa (XLW)	5700–5200	72	1033	32.103
Zhaobaogou (ZBG)	5200–4500	24	561	12.197
Hongshan (HS)	4500–3000	15	324	5.679
Lower Xiajiadian (LXJD)	2000–1200	105	2366	60.809
Sum		216	4572	117.512 (1.175 km^2^)

**Table 2:** Model results of five subsequent periods (all parameters).

	XHX	XLW	ZBG	HS	LXJD
Observations	15	72	24	15	105
% land cover	0.19	0.26	0.14	0.15	0.37
ROC AUC	0.88	0.82	0.93	0.91	0.80
Test gain	1	0.85	2.2	1.61	0.88

**Table 3:** Xiaohexi (XHX) model results: Important parameters sorted by their relative contribution.

Xiaohexi.		
	Test Gain	Land Cover (%)
**Aspect**	0.36 *	**36.9%**
**Forest Dist.**	0.31 *	**45.2%**
Modern Built Distance	0.16	62%
River Dist.	0.12	62%
Crop Dist.	0.21	66%
Slope	0.01	72%
Elevation	0.004	80%
Land Curvature	-0.04	88%

* top contributors.

**Table 4:** XLW model results: important parameters sorted by their relative contribution.

Xinglongwa		
	Test Gain	Land Cover (%)
**Aspect**	0.39 *	**38%**
Forest Dist.	0.06	66%
Modern Built Distance	0.06	70%
River Dist.	0.007	82%
**Crop Dist**	0.25 *	**65%**
Slope	0.06	60%
Elevation	0.01	74%
Land Curvature	0.11	66%

* top contributors.

**Table 5:** ZBG model results: important parameters sorted by their relative contribution.

Zhaobaogou		
	Test Gain	Land Cover (%)
**Aspect**	0.6 *	**41%**
Forest Dist.	0.6	70%
Modern Built Distance	0.11	60%
**River Dist.**	0.32 *	**45%**
Crop Dist.	0.25	60%
Slope	0.14	50%
Elevation	0.21	48%
Land Curvature	0.16	60%

* top contributors.

**Table 6:** HS model results: important parameters sorted by their relative contribution.

Hongshan		
	Test Gain	Land Cover (%)
**Aspect**	0.41 *	**49%**
Forest Dist.	0.03	65%
Modern Built Distance	0.12	73%
River Dist.	0.16	57%
Crop Dist.	0.16	69%
Slope	0.04	58%
Elevation	0.17	64%
Land Curvature	0.19	56%

* top contributors.

**Table 7:** LXJD model results: important parameters sorted by their relative contribution.

Lower Xiajiadian		
	Test Gain	Land Cover (%)
Aspect	0.10	65%
Forest Dist.	0.17	66%
Modern Built Distance	0.14	58%
River Dist.	0.11	77%
Crop Dist	0.18	65%
Slope	0.07	67%
Elevation	0.01	66%
Land Curvature	0.11	57%

**Table 8:** Model results of the five successive periods, sorted by important parameters and their relative contribution according to the MaxEnt threshold values which predict 80%, 85%, and 90% of the collection units, respectively.

**80% of** collection units **threshold**	**XHX**		**XLW**		**ZBG**		**HS**		**LXJD**	
	**Test gain**	**Land cover (%)**	**Test gain**	**Land cover (%)**	**Test gain**	**Land cover (%)**	**Test gain**	**Land cover (%)**	**Test gain**	**Land cover (%)**
*Aspect*	0.33 *	36.5%	0.27 *	36%	0.6 *	26%	0.43 *	43%	0.08	59%
*Forest Dist.*	0.31 *	42.%	0.06	65%	0.62	70%	0.03	65%	0.14	66%
*Modern Built Dist.*	0.1	61%	0.04	66%	0.13	40%	0.17	62%	0.10	56%
*River Dist.*	0.12	61%	0.001	81%	0.29 *	42%	0.11	56%	0.15	58%
*Crop Dist.*	0.21	66%	0.25 *	64%	0.25	60%	0.17	65%	0.15	64%
*Slope*	0.05	60%	0.06	50%	0.14	50%	0.04	55%	0.10	48%
*Elevation*	0.004	78%	0.01	74%	0.22	45%	0.19	60%	0.02	63%
*Land Curvature*	-0.04	65%	0.08	60%	0.19	57%	0.20	40%	0.11	52%
**85% of** collection units **threshold**	**XHX**		**XLW**		**ZBG**		**HS**		**LXJD**	
	**Test gain**	**Land cover (%)**	**Test gain**	**Land cover (%)**	**Test gain**	**Land cover (%)**	**Test gain**	**Land cover (%)**	**Test gain**	**Land cover (%)**
*Aspect*	0.36 *	36.9%	0.39 *	38%	0.6 *	41%	0.41 *	49%	0.10	65%
*Forest Dist.*	0.31 *	45.2%	0.06	66%	0.6	70%	0.03	65%	0.17	66%
*Modern Built Dist.*	0.16	62%	0.06	70%	0.11	60%	0.12	73%	0.14	58%
*River Dist.*	0.12	62%	0.007	82%	0.32 *	45%	0.16	57%	0.11	77%
*Crop Dist.*	0.21	66%	0.25 *	65%	0.25	60%	0.16	69%	0.18	65%
*Slope*	0.01	72%	0.06	60%	0.14	50%	0.04	58%	0.07	67%
*Elevation*	0.004	80%	0.01	74%	0.21	48%	0.17	64%	0.01	66%
*Land Curvature*	-0.04	88%	0.11	66%	0.16	60%	0.19	56%	0.11	57%
**90% of** collection units **threshold**	**XHX**		**XLW**		**ZBG**		**HS**		**LXJD**	
	**Test gain**	**Land cover (%)**	**Test gain**	**Land cover (%)**	**Test gain**	**Land cover (%)**	**Test gain**	**Land cover (%)**	**Test gain**	**Land cover (%)**
*Aspect*	0.30 *	39.0%	0.40 *	40%	0.62 *	42%	0.42 *	49%	0.10	77%
*Forest Dist.*	0.31 *	45.3%	0.04	67%	0.56	69%	0.03	65%	0.26	69%
*Modern Built Dist.*	0.08	71.8%	0.07	76%	0.13	70%	0.14	74%	0.11	67%
*River Dist.*	0.11	64%	0.007	85%	0.29 *	59%	0.16	57%	0.11	77%
*Crop Dist.*	0.22	67%	0.25 *	66%	0.3	63%	0.17	73%	0.19	65%
*Slope*	0.04	72%	0.06	65%	0.14	60%	0.04	75%	0.07	77%
*Elevation*	0.003	82%	0.02	75%	0.24	66%	0.17	78%	0.01	78%
*Land Curvature*	-0.03	83%	0.12	69%	0.19	59%	0.19	72%	0.10	76%

* top contributors.

## Supplementary Materials

The following are available online at https://www.mdpi.com/1099-4300/22/3/307/s1. S1. Supplementary data and code scripts; S2. Fuxin regional survey—collection units and numeric data; S3. Autocorrelation test between predictor variables.
