# Supplementary figures and images for: Using the Maximal Entropy Modeling Approach to Analyze the Evolution of Sedentary Agricultural Societies in Northeast China
Source: Entropy (Basel). 2020 Mar 9;22(3):307. doi: 10.3390/e22030307 (PMC7516762; doi:10.3390/e22030307)

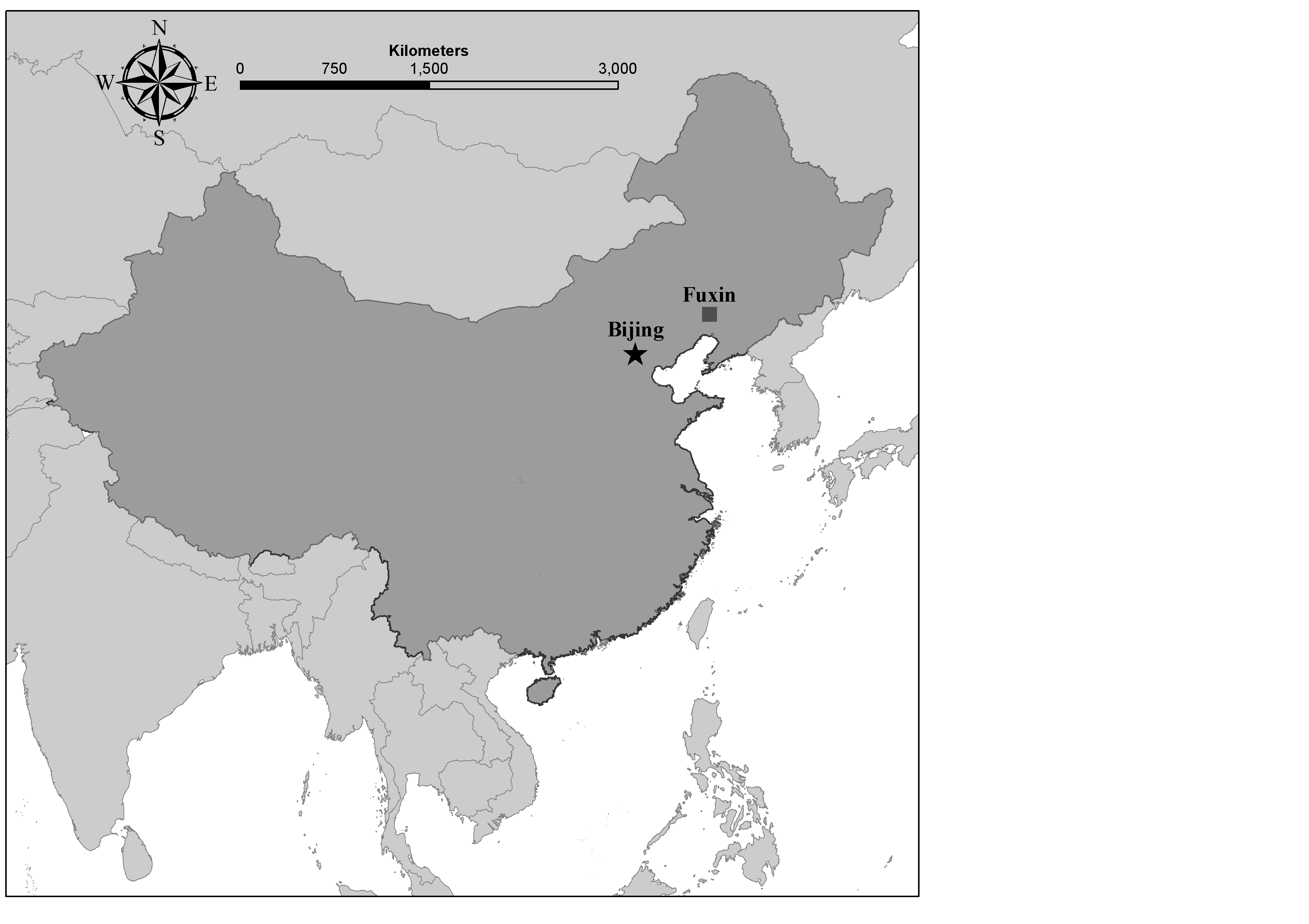

Supplement: Supplementary file 1 [file entropy-22-00307-s001.zip › Figures_600dpi_Round3/Figure 1 The location of the Fuxin area in China.tif]

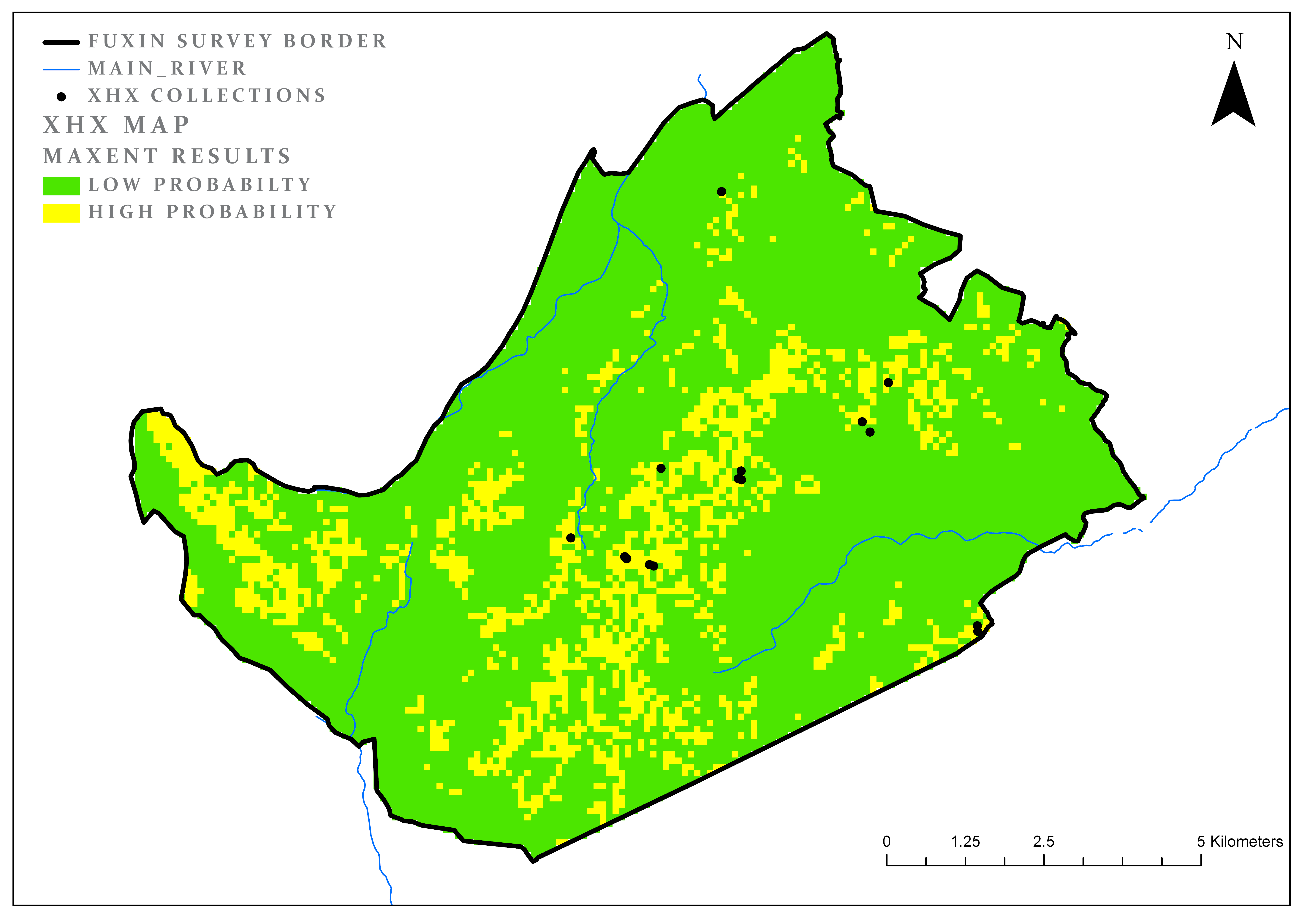

Supplement: Supplementary file 1 [file entropy-22-00307-s001.zip › Figures_600dpi_Round3/Figure 2. XHX model results.tif]

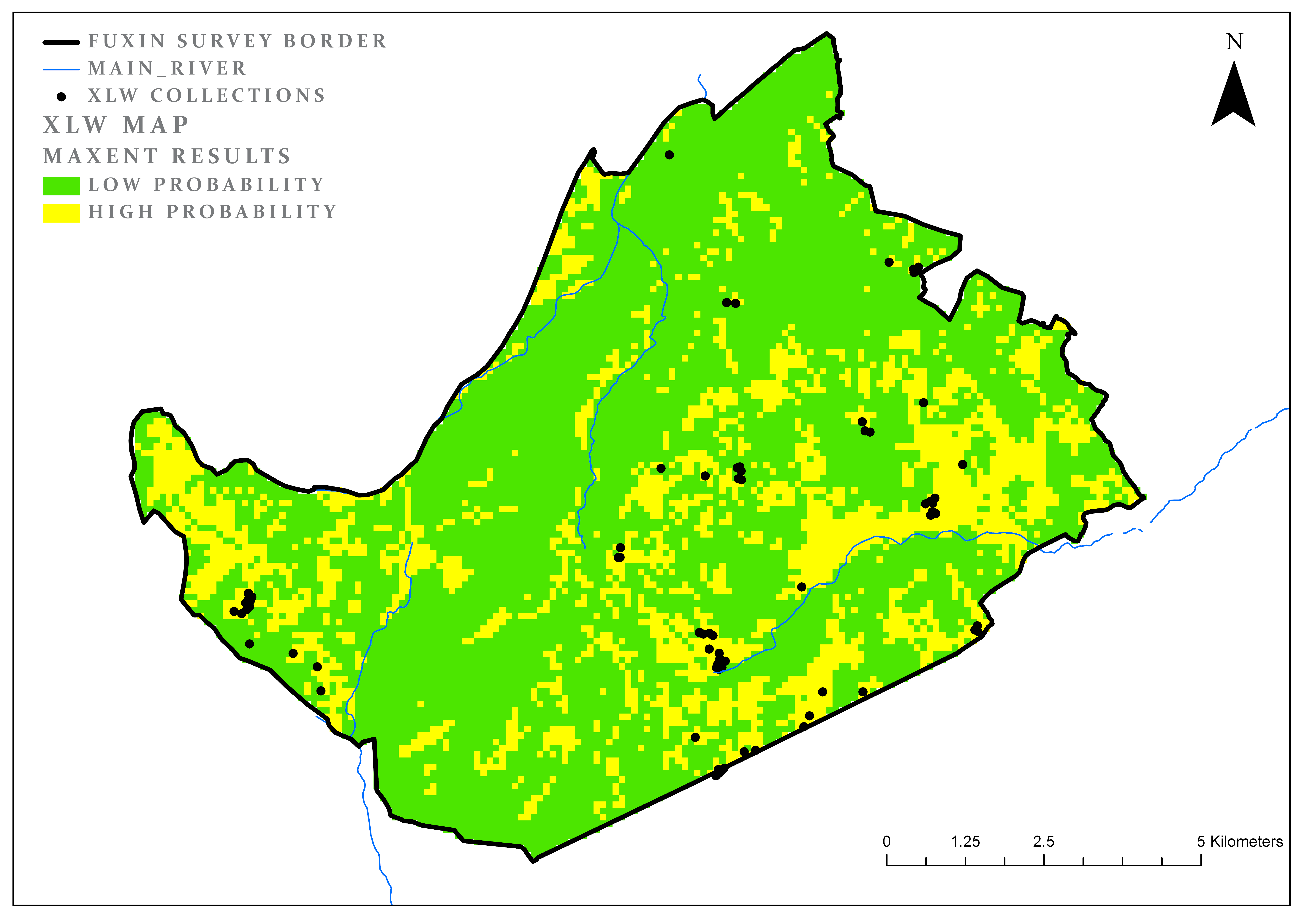

Supplement: Supplementary file 1 [file entropy-22-00307-s001.zip › Figures_600dpi_Round3/Figure 3. XLW model results..tif]

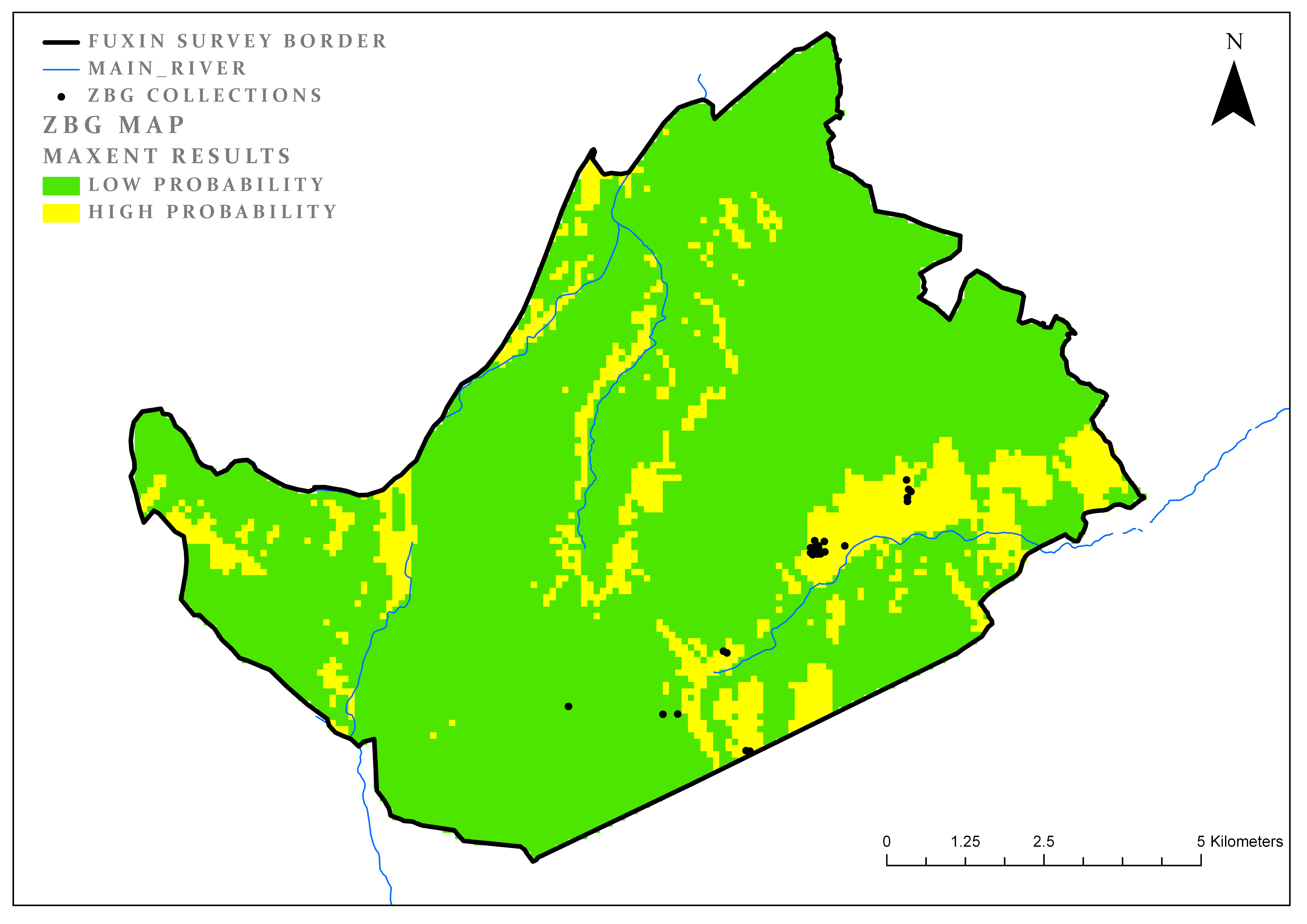

Supplement: Supplementary file 1 [file entropy-22-00307-s001.zip › Figures_600dpi_Round3/Figure 4. ZBG model results.tif]

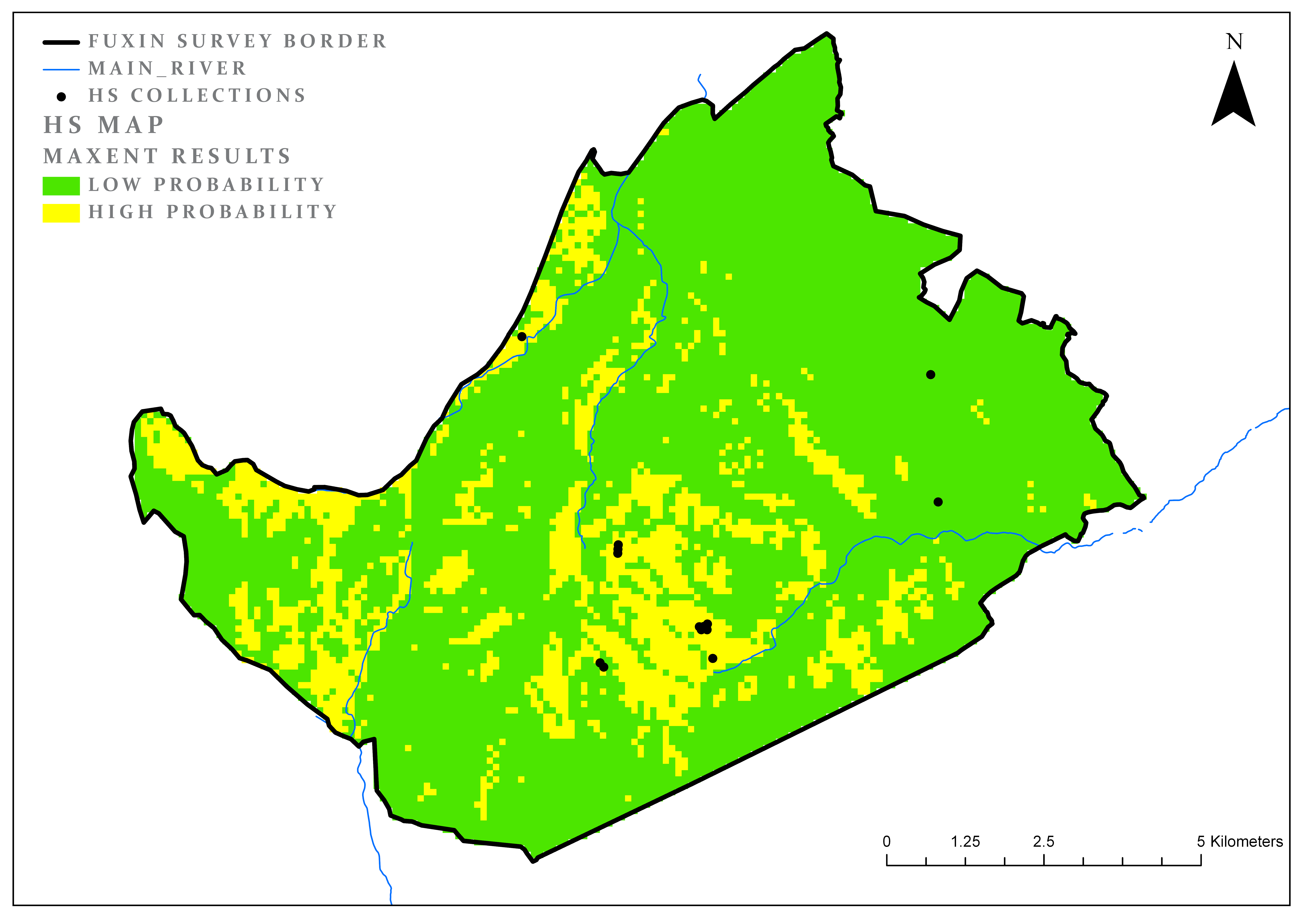

Supplement: Supplementary file 1 [file entropy-22-00307-s001.zip › Figures_600dpi_Round3/Figure 5. HS model results.tif]

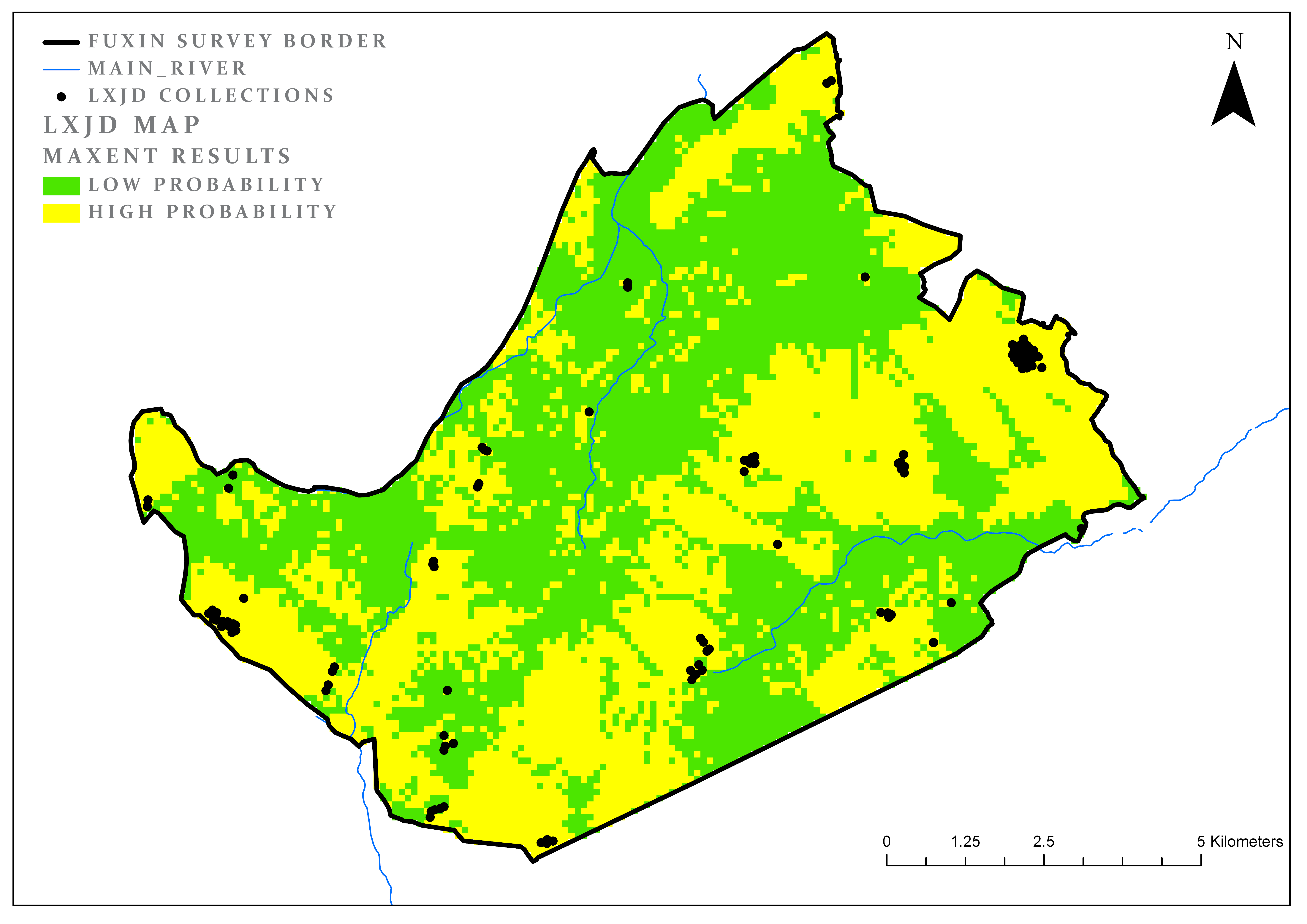

Supplement: Supplementary file 1 [file entropy-22-00307-s001.zip › Figures_600dpi_Round3/Figure 6. LXJD model results.tif]
